# Macroautophagy and the Oncogene-Induced Senescence

**DOI:** 10.3389/fendo.2014.00157

**Published:** 2014-09-29

**Authors:** Daniel Grasso, Maria I. Vaccaro

**Affiliations:** ^1^Institute of Biochemistry and Molecular Medicine, National Council for Scientific and Technological Research, Department of Pathophysiology, School of Pharmacy and Biochemistry, University of Buenos Aires, Buenos Aires, Argentina

**Keywords:** senescence, oncogene-induced senescence, autophagy, tumor suppression mechanisms, cancer

## Abstract

The oncogene-induced senescence is emerging as a potent tumor suppressor mechanism and as a possible therapeutic target. Macroautophagy is intimately linked to the senescence condition setup, although its role has not been elucidated yet. Here, we discuss up-to-date concepts of senescence-related macroautophagy and evaluate the current trend of this growing research field, which has relevance in future perspectives toward therapeutic options against cancer.

## Introduction

Macroautophagy is a catabolic process for the recycling and degradation of cellular constituents (1–3). The implication in several physiological and pathological processes, as well as in basic cellular mechanisms, has made macroautophagy one of the hot research topics of the last decade. The increased interest in macroautophagy has led to a deeper understanding of its molecular mechanisms, unmasking it as a process with unthinkable roles. Macroautophagy sequesters and degrades portions of cytoplasm including entire organelles; this is termed bulk macroautophagy (2–4). In this way, macroautophagy is an effector process of nutrient states sensing factors, principally mTOR (mammalian Target Of Rapamycin) and AMPK (AMP-activated Protein Kinase) (5). The mTORC1 (mTOR Complex-1) is composed by RAPTOR and DEPTOR, two negative regulators, mLST8, a positive regulator, and PRAS40 (6). With the Ser/Thr protein kinase activity of mTOR, the mTORC1 is a key integrator of the nutrient status and nutrients availability, growth factors, and stress signals (6). In amino acid starvation, mTORC1 is inactivated and distributed dispersed into the cytoplasm. Upon amino acid stimulation, mTORC1 is redistributed mainly to lysosomal membranes where, in response to amino acids, RAG GTPases activate the complex (6). Finally, the plenty nutrient state activates the mTORC1, which in turn induces cell growth and protein synthesis, and inhibits catabolic processes such as macroautophagy (5, 6).

The first link between the nutritional cell condition and macroautophagy is the protein kinase Ulk1 (Unc-51 like kinase-1), or its homolog Ulk2 (5). Ulk1 forms a complex with Atg13 and FIP200 that enhances the activity of the protein kinase Ulk1 (5). Activated mTORC1 maintains Atg13 and Ulk1 in a hyperphosphorylated state, avoiding the triggering of the macroautophagy. In starvation, the inactivation of mTORC1 leads to the hypophosphorylation of these proteins, releasing and activating the Ulk1 complex. Although the substrate of Ulk1 is so far unknown, the kinase activity of this complex is able to initiate the macroautophagy cascade (5).

**Figure 1:**
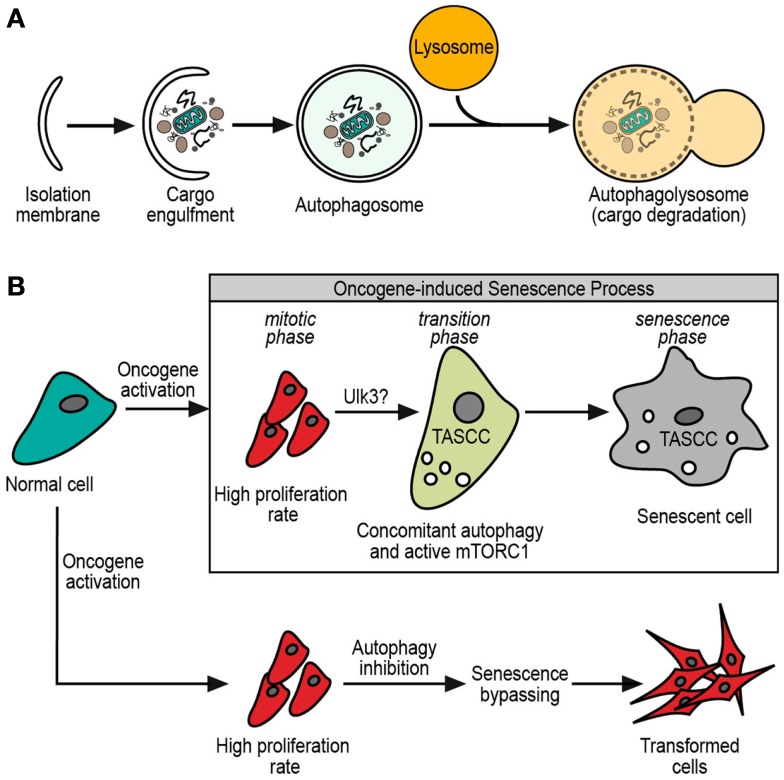
**(A)** General scheme of macroautophagy process. The initial isolation membrane is grown by lipids and macroautophagy-related proteins in order to engulf the cargo target. Then, the fusion of the isolation membrane edges forms a double membrane vesicle, termed autophagosome. The autophagosome eventually fuses with lysosome, resulting in the autophagolysosome. Finally, it is in the autophagolysosome where cargo and autophagosome inner membrane are degraded by the lysosomal enzymes. **(B)** Normal cell after oncogene activation. After oncogene activation, cells have at least two possible fates. One of them is an oncogene-driven transformation where senescence must be bypassed, and macroautophagy inhibition could contribute to this bypassing. The other option is to enter in an oncogene-induced senescence with a permanent cell cycle arrest. During oncogene-induced senescence, three phases are observed: the *mitotic phase*, with high proliferation rate; the *transition phase*, with the possible participation of Ulk3 in the macroautophagy triggering and apparition of TASCC (see text); finally, the *senescence phase* with the definitely senescence phenotype established.

Structurally, macroautophagy starts with the *omegasome*, which is a structure that sprouts from the ER membrane, and grows by lipids and several macroautophagy proteins (2, 3). This budding-like process gives initiation to the *phagophore*, a tiny vesicle precursor of the *isolation membrane* that is formed by the growing of the phagophore with more lipids and autophagy-related proteins (ATG proteins) (7). This bigger membrane eventually engulfs the cargo, forming a double membrane vesicle termed the *autophagosome*. The outer membrane of the autophagosome fuses with lysosomes for the delivery of its inner vesicle and cargo into a degradative organelle (the *autophagolysosome*) (7) (Figure 1A).

The deeper insight into these phenomena has revealed more specialized and highly controlled forms of macroautophagy. Although all types of macroautophagy may share the same core molecular machinery involved in the autophagosome formation, today we recognize conceptually and mechanistically several forms of selective macroautophagy such as mitophagy, pexophagy, ribophagy, lipophagy, and zymophagy (8, 9). Furthermore, the macroautophagy field has also expanded its implication in physiological and pathological processes, such as life span, starvation, embryogenesis, cancer, degenerative diseases, Crohn’s disease, pancreatitis, and host defense.

## Macroautophagy in Cancer

The role of macroautophagy in cancer is quite conflictive and confusing (10). In mammals, it has been linked to tumor development, since one of the most important macroautophagy-related molecules, Beclin 1 (a Bcl2-interacting protein), is a haploinsufficient tumor suppressor gene (11, 12). Indeed, in Beclin 1 + ∕− transgenic mice, that display reduced macroautophagy levels, a significant increase of spontaneous tumor incidence is observed (13). Several reports present macroautophagy and macroautophagic cell death as anti-tumoral responses. On the other hand, macroautophagy is related to chemotherapeutics resistance (14, 15) and to the enhancement of cancer-cell metabolism during hypoxic and nutrient-deficient environments (16).

## Oncogene-Induced Senescence

The term cellular senescence is used to describe a deep and irreversible arrest status of the cell cycle. In this process, cells suffer a radical transformation with a strong repression of proliferation genes that eventually cause an increase in heterochromatin areas into the nucleus, loss of response to growth factors, and also a deep modification in cell metabolism and morphology including an expanded and flattened cytoplasm shape, and increase of focal adhesions among others (17). Lysosomal enzyme β-galactosidase activity at pH 6.0 is the hallmark and the most spread assay for senescence determination, the so-called senescence-associated β-galactosidase (SA-βGal). In spite of this, the SA-βGal assay is not fully specific for senescence, since it could be positive also in other cellular conditions (18). Consequently, a conjunction of markers should be used to evaluate senescence more accurately. Among such markers are: the cell morphology, the presence of SAHFs (Senescence-Associated Heterochromatic Focis), DNA damage response (DDR) markers (i.e., γ-H2AX, CHK2,) and the evaluation of p53, p16*^INK4a^*, and p21*^CIP1^*.

Curiously, the senescence is a common output response to different cellular stressors. One of them is shortened telomeres caused by the successive DNA replications. This type of senescence is termed *replicative senescence* (sometimes abbreviated as RS), and was first described by Hayflick in 1961 (19), who demonstrated that cells possess a limited number of replications by means of successive passage of human diploid fibroblasts. In this setting, the unprotected telomeres are interpreted as double strand breaks of DNA and trigger the cellular DDR, which eventually activates the cellular arrest mechanisms (20). The final effector pathways are through the p53/p21*^CIP1^* and p16*^INK4a^* axes, leading to the hypophosphorylated form of Rb, which in turn represses the activity of cell cycle progression-related transcription factors (20). Furthermore, the agents or events that lead to DNA damage, such as UV light, chemotherapeutics, and ROS, are capable of triggering a premature senescence in the cell (21, 22). This last event, is commonly entitled as *stress-induced premature senescence* (sometimes abbreviated as SIPS), and also activates the DDR system in order to arrest the cell cycle in a telomere-independent fashion. Finally, the senescence program can be additionally triggered by the activation of an oncogene, termed *oncogene-induced senescence* (sometimes abbreviated as OIS) (21, 22). The oncogene-induced senescence is also independent of telomeres and can be mediated by the DDR program. It could be possible that the initial high replication rates, induced by oncogene activation, results in DNA damage, a process where the ROS generation could also be implicated (21, 22). Nevertheless, the oncogene-induced senescence is also able to trigger senescence independently of DDR activation, by induction of the p53/p21*^CIP1^* or p16*^INK4a^* pathways, but the mechanism is not fully understood (20). Finally, these differences are clearly appreciated in recent data that compare gene expression levels between the replicative senescence and the oncogene-induced senescence. Although the final consequence is the same, meaning the senescence phenotype, there are deep differences in the genetic programs implicated in both cases (23).

The first description by Hayflick (19) promoted the idea that senescence probably was just an *in vitro* artifact. However, as more data are being obtained, senescence is recognized as a true cellular “*aging*” program (17, 24). Concerning the impact of cellular senescence to the whole organism, Martin et al. (25) and Goldstein et al. (26) found an impairment in the replicative capacity of fibroblast from accelerated aging syndromes and age-related human diseases. However, there is not enough evidence to relate the duplication capacity of fibroblasts with the lifespan of the organisms (27). Recent interesting findings in the field show that senescence is a key cellular pathway (28), which may function as an alternative mechanism to apoptosis in response to certain stressors. Thereby, the oncogene-induced senescence appears to play a role as a tumor suppressor mechanism against cellular transformation (29–32). The oncogene-induced senescence has been reported in several murine (29) and human (29) tumors; and is believed to be one of the first cellular barriers against transformation (30, 33). One excellent example is the results reported by Guerra et al. (33) using a murine model of pancreatic carcinogenesis. The transgenic expression of the oncogenic Kras is only able to induce low grade pre-cancerous structures into the pancreatic tissue. These structures are highly positive for different senescence markers and only after the induction of pancreatitis, senescence can be overcome and the model progresses toward tumor development (33). Upon oncogene activation, cells have three principal outcomes: to proliferate; to trigger an oncogene-induced apoptosis; or to enter in an oncogene-induced senescence process (29, 30). One example is the K562 leukemia cells in which the *imatinib* treatment induces massive apoptosis with low percentage of cells entering in senescence; but this percentage of senescent cells is highly increased when apoptosis is inhibited (34). Therefore, one of the events that cells can induce against oncogene activation is the senescence, which apparently is triggered by the oncogene itself. This senescence induction by oncogene activation and the requirement to bypass the tumor suppressive process in order to transform cells was clearly demonstrated by Pérez-Mancera and Tuveson in 2006 (35). They infected mouse embryonic fibroblasts (MEFs) with a retrovirus carrying the oncogenic Ras, inducing a supraphysiological oncogene expression (35). In that experiment, the logical expectation is MEFs transformation, but instead of that, MEFs surprisingly entered in an early senescence process (35). More interestingly, when they activated a pre-existing LSL-Kras^G12D^ allele by a Cre recombinase virus, they obtained physiological Kras^G12D^ expression levels and a partial transformation was reached (35). All these data suggest that oncogene-induced senescence is a kind of failsafe mechanism. For example, cells respond to growth factors with proliferation, but when things go out of control, senescence appears into the game.

Despite the differences among cell types, a sequence of steps is observed during the establishment of the oncogene-induced senescence (27). This process consists in an initial response to the oncogene activation, which leads to hyperproliferation, known as *mitotic phase*. Then a *transition phase*, where the proliferation slows down and senescence characteristics start to be evident. Finally, a *senescence phase*, where the process is widely established (Figure 1B). Although there was significant progress in the past years, the complete details about the oncogene-induced senescence mechanisms remain to be elucidated.

## Macroautophagy as Senescence Effector

The pioneering works by Bergamini et al. (36, 37), early suggested a relationship between macroautophagy and aging. Following publications strengthen those data, linking macroautophagy to senescence-related events. Furthermore, recent works strongly support the key role of macroautophagy in the oncogene-induced senescence process. During senescence, there is a gradual increase in Bag3/Bag1 ratio inducing a cellular switch from proteasome to macroautophagy for polyubiquitinated proteins degradation (38). This phenomenon is also observed in tissue aging (38). In addition, it was suggested that the macroautophagy pathway during the oncogene-induced senescence has the ability to process chromatin, contributing to stability of senescence and tumor suppression (39).

Macroautophagy was found several times during senescence in the transition phase (40), but it was not until the work of Young et al. (41) that macroautophagy was described as a senescence effector. They observed an increased macroautophagy level in IMR90 cells entering in senescence by Hras^V12^ retroviral transduction, which is not observed in quiescent cells (41). They documented that senescence led to a high coupling synthesis-degradation with an apparently paradoxical situation, where the same cell presents high macroautophagy activity with the autophagy inhibitor, mTOR, activated at the same time (41). This situation was explained by the outstanding publication by Narita et al. (42), with the introduction of the *TOR-autophagy spatial coupling compartment* (TASCC) concept. They described an amazing cytoplasmic sub-compartmentalization, the TASCC, where cell literally hide mTORC1 from macroautophagy machinery in order to avoid its inactivation (42). In TASCC, mTOR is closely associated with lysosomes/autolysosomes, fueled by a constant macroautophagy flow outside this area (42). In this way, the amino acids released by lysosomal degradation maintain mTOR activation, thereby sustaining a high protein synthesis level (42). Moreover, this synthesis–degradation coupling through the TASCC seems to be important in the physiology of some specialized cells, such as the kidneys’ podocytes that have the constitutive need to phagocyte and degrade the glomerulus basal membrane and at the same time maintain protein synthesis (42, 43). This opens the possibility that TASCC can additionally play other physiological functions. For example, recently we described the important role of zymophagy (selective macroautophagy of activated zymogen granules) to prevent intracellular activation of zymogen granules in pancreatic acinar cells (8). These cells have a metabolism almost completely dedicated to protein synthesis to accomplish their function (44). Therefore, we can hypothesize that the TASCC strategy can allow the zymogen synthesis and at the same time the prophylactic basal zymophagy activity. Nevertheless, whether the TASCC has always the same composition and function remains unknown.

## Role of Macroautophagy in Senescence

It is interesting that macroautophagy seems to be very important to the senescence process, but its role seems to depend on the cellular context and the experimental settings. In *normal cells*, the 3-methyladenine-mediated inhibition of macroautophagy produces a significant attenuation in the premature senescence developed by HUVEC cells exposed to glycated collagen I (45). In this work, it is also suggested that, at least in this case, macroautophagy is triggered by the lysosomal membrane permeabilization as cellular stress response, which in turn leads to a senescence phenotype (45). Lysosomes are the final destination of autophagosomes; hence, a dysfunction of these organelles impairs the autolysosome formation and consequently the autophagic flow. In this way, Patschan et al. (45) observed that the lysosomal membrane permeabilization induces macroautophagy and triggers a stress-induced premature senescence, which is reversible by autophagy inhibition. Altogether, these data reveal a profound relationship between the macroautophagy processes and senescence.

In *immortalized cells*, Young et al. (41) showed that the adenoviral oncoprotein E1A expression suppresses the Ras-induced senescence and significantly reduces the macroautophagy and protein degradation. These data suggest that macroautophagy contributes to oncogene-induced senescence. Singh et al. (46) further support this concept by showing that chronic expression of a proteolytic Cyclin E fragment induces autophagy and senescence. Furthermore, senescence is prevented by macroautophagy inhibition and in turn, apoptosis is triggered (46). What is more, induced senescence in that setting is Atg7-dependent (46). In the work of Drullion et al. (34), commented above, a significant reduction of SA-βGal positive cells was observed upon downregulation of Atg7 or Beclin 1. Very interestingly, is the fact that conversely to Atg7, the depletion of Atg5 induces an increased number of SA-βGal positive cells (34).

In the case of *transformed cells*, the retrovirus-mediated Hras*^V12^* transduction in diverse human diploid fibroblasts cells (IMR90, BJ, or embryonic skin fibroblasts) produces an increase in macroautophagy levels and a subsequent senescence (41). In those experiments, the shRNA-mediated depletion of Atg5 or Atg7 attenuates the Ras-activated autophagy and delays the SA-βGal in IMR90 cells. Moreover, in BJ cells, the downregulation of one or the other autophagic-related proteins is enough to bypass senescence (41). Dissimilar results come from the work of Wang et al. (47). They show that ASPP2 (apoptosis-stimulating of p53 protein 2), through its Nt-domain, inhibits macroautophagy and in this fashion induces oncogene-induced senescence in MEFs transduced with the oncogenic Hras^V12^ (47). Furthermore, similar to the results by Drullion et al. (34) about Atg5, Wang observed that under Atg5 overexpression, cells are able to bypass the Ras-induced senescence. On the contrary, the depletion of Atg5 or Atg3, under the oncogenic Ras expression, condemns the cells to senescence (47). Altogether, these data suggest that despite some discrepancies, macroautophagy plays a key role in the establishment of the senescence phenotype. What is more interesting is that according to these findings, Atg5 and Atg7 might play opposite roles in senescence. They are both very important molecules implicated in the macroautophagy, but their antagonism in the senescence induction may suggest a finely tuning of the macroautophagy-associated senescence. However, further work is needed to fully understand this process.

Working with chemoresistant cells from tumor tissue, Nam et al. (48, 49) observed that prolonged macroautophagy, due to mTOR inhibition, triggers senescence (48, 49). In this case, the mTOR inhibition seems to have key role. However, we have to take into account that different to aforementioned data, cells are already transformed. Of note, this is strengthened by xenograft experiments, where the rapamycin (mTOR inhibitor) treatment increases macroautophagy and SA-βGal positive staining, and delays tumor growth (48, 49). Dissimilar results are also obtained in iPSCs (induced pluripotent stem cells) with a senescence dependent in mTOR activity (50). In this experimental model, rapamycin treatment inhibits mTOR activity, and the consequent induction of macroautophagy leads cells to prevent senescence and promote reprograming (50). Here, again we have discrepancies in the role of autophagy in senescence. Although they are quite different in experimental situations, in both of them we have a rapamycin treatment, which inhibit mTOR activity with the consequent increase in macroautophagy activity. In this apparently similar experimental context, the increased macroautophagy is pro-senescence in the first experience and anti-senescence in the second. Again, we could speculate about different macroautophagy mechanisms such as different actions of Atg5 and Atg7, commented before. However, we cannot discard that these discrepancies could originate from differences in the intracellular contexts.

Another possible explanation to the different roles of macroautophagy in senescence could be that other/s pathway/s must complement it, in order to reach one or another outcome. The *geroconversion* is the term used to describe the conversion of a cell from a quiescent state, a reversible arrest, to senescence, which is an irreversible arrested state (51). Working with normal, transformed, and immortalized cells, Leontieva et al. (51) demonstrated that cells in hypoxic conditions are more resistant to senescence induction. The hypoxia increases macroautophagy and inhibits mTOR activity, suppressing the geroconversion caused by diverse stimuli (51). We have already mentioned the role of TASCC during the oncogene-induced senescence, where the cytoplasmic sub-compartmentalization allows an operational macroautophagy flux with an activated mTOR at the same time. Therefore, we could speculate that macroautophagy is enough to reach a cell’s state of quiescence, but the simultaneous mTOR activity is needed as complement to arrive to the senescence phenotype. Additionally, also supporting this idea, Lerner et al. (52) show that a chronic rapamycin treatment extends cell lifespan, in a telomere-independent fashion of quiescent normal fibroblasts. They described that chronic rapamycin-mediated inhibition of mTOR, in those quiescent cells leads to an increase in mitochondrial mass and biogenesis, reduction of ROS (reactive oxygen species) and macroautophagy induction, which in turn suppresses senescence (52). Understanding the mechanisms behind these last concepts is of fundamental importance for future approaches of cancer therapeutics, more precisely in resolving the tumor cells dormancy. Clinically, tumor dormancy explains the observations that cancer patients can relapse years to decades after an apparently successful treatment. In these situations, tumor cells remain at the primary site or in distant metastases, which are causative of major cancer-related deaths. In cell dormancy, the role of oncogene pressure over the cell as well as the cellular microenvironment is of great importance (53). It has been demonstrated that after some genetic and epigenetic modifications, tumor cells can become oncogene addicted (53). In those cases, oncogene inactivation can induce quiescence, differentiation, apoptosis, or senescence (53). Moreover, unfavorable microenvironments can also challenge the tumor cell into stressful conditions (54). In those scenarios, autophagy plays a fundamental role in giving a survival opportunity and maintaining a quiescent cell state (54). All these data expose autophagy as double-bladed sword since it can contribute to senescence, in an irreversible cell cycle arrest that could be eliminated by the immune system, or it can favor the cell toward quiescence in a dormancy state and future relapse of disease. Different strategies in order to manipulate macroautophagy to favor senescence over the quiescence must to be evaluated.

The description of specialized forms of macroautophagy in eukaryotic cells, tempts us to hypothesize that there might be a senescence-specific type of macroautophagy. The main controversy about the role of macroautophagy in senescence comes from the facts described above, where sometimes it is pro-senescent and sometimes anti-senescent. We have already mentioned the differences between Atg5 and Atg7 suggesting some unrelated roles of these proteins in the senescence-related macroautophagy. One small but interesting finding came from the work of Young et al. (41), where canonicals Ulk1/2 protein kinases do not trigger senescence-related macroautophagy. Instead, senescence is triggered by Ulk3 (Figure 1B), which shares 31.6% of homology with Ulk1/2 (5). Moreover, Ulk3 is able to colocalize with Atg12-5-16 complex and its overexpression is sufficient to trigger macroautophagy and senescence (41). These findings show that senescence-related macroautophagy might have different molecular mechanisms. Taking into account that three homologs of Ulk1/2 have been described, alternative mechanisms would be possible (5). Further investigation is necessary to understand the molecular relationships between macroautophagy and senescence.

Finally, another aspect is the time of evaluation. In some cases, macroautophagy is evaluated before and in others after the transition phase of senescence. In the first situation, macroautophagy may contribute to senescence establishment, acting as a tumor suppressor mechanism. However, once senescence is bypassed, macroautophagy shows its dark side enhancing metabolic stress resistance and even chemotherapeutic resistance of transformed cells. Therefore, macroautophagy may have different functions depending on the time of evaluation and more importantly, these functions may involve different types of macroautophagy.

## Senescence-Associated Secretory Phenotype

The senescence-associated secretory phenotype or SASP is a secretory complex mix of proteins and molecules in which senescent cells invest a lot of energy. It is composed, at least in part, by pro-inflammatory cytokines, chemokines, and tissue remodeling enzymes (55). It is believed that SASP induces some inflammatory response and the clearance of senescent cells, although it could also enhance malignancy (55, 56). In addition, SASP possesses a paracrine effect, inducing and reinforcing senescence phenotype in neighboring cells (56). Whether SASP is also able to induce macroautophagy remains unclear.

On the other hand, macroautophagy actively participates in SASP, since TASCC is fundamental to maintain the synthesis and the release of these molecules in senescent cells (42, 57). In this way, macroautophagy induction could favor the tissue response to tumoral transformation. In recent years, the idea of a therapeutic-induced senescence as a potential cancer treatment has gained importance (30, 58, 59). However, that idea has a double edge, since senescence induction could derive in tumor cell dormancy and cancer relapse (60, 61).

## Conclusion

In the recent years, a significant progress has been made in the molecular mechanisms that link macroautophagy to senescence, although, it seems to be the tip of the iceberg. The future elucidation of the complete landscape surely will impact strongly in our knowledge of cancer, its treatment, our vision of cell’s tumor suppressive strategies, but it will also have important implications in several physiological and pathophysiological processes. Such is the case of TASSC description that seems to have non-senescence-related physiological roles. These facts, in addition to the control of oxidative stress by mitophagy or the protective role of zymophagy in acinar cells, among other examples, strongly change our concept of macroautophagy from a general catabolic process to a highly specific programed cellular process. This point of view shows macroautophagy plasticity to make simple works such as the cytoplasm bulk degradation to highly regulated, and indeed compartmentalized in the TASSC, senescence-related macroautophagy. Finally, the study of senescence-related macroautophagy will reveal an unthinkable plethora of new knowledge and, at the same time, will impact in therapeutic strategies against complex human diseases.

## Conflict of Interest Statement

The authors declare that the research was conducted in the absence of any commercial or financial relationships that could be construed as a potential conflict of interest.
